# Parental Acceptance of Telemedicine in Pediatric Surgery and Its Implications for Future Care Models: Survey Study

**DOI:** 10.2196/81091

**Published:** 2026-02-23

**Authors:** Nariman Mokhaberi, Sara Peggion, Safiullah Najem, Konrad Reinshagen

**Affiliations:** 1 Department of Pediatric Surgery, University Medical Center Hamburg-Eppendorf, Martinistr. 52, Hamburg, 20251, Germany, 49 4088908 ext 173; 2 Department of Pediatric Surgery, Altonaer Kinderkrankenhaus, Hamburg, Germany; 3 Department of Pediatric Surgery, Heinrich Heine University Düsseldorf, Düsseldorf, Germany; 4 Department of Pediatric Surgery, Medical University of Vienna, Vienna, Vienna, Austria; 5 German Center for Child and Adolescent Health (DZKJ), Partner Site Hamburg, University Medical Center Hamburg-Eppendorf, Hamburg, Germany

**Keywords:** telemedicine, pediatric, pediatric surgery, digitalization, telehealth

## Abstract

**Background:**

Digitalization has profoundly transformed health care delivery, including the increasing use of telemedical applications in pediatric care. While the economic benefits and improved access associated with telemedicine in rural regions are well documented, data on caregiver acceptance and demand in metropolitan areas remain limited.

**Objective:**

This study aimed to assess caregivers’ willingness to use telemedical tools in the context of pediatric surgery in a metropolitan area.

**Methods:**

A 15-item survey was distributed to caregivers of pediatric surgical patients between May and November 2023 at the Department of Pediatric Surgery of the University Medical Center Hamburg-Eppendorf. The survey included questions on sociodemographic factors, previous experience with telemedicine, and preferences regarding telemedical support. Data were analyzed using descriptive statistics. Group comparisons were performed using the *χ*^2^ test or Fisher exact test, where appropriate.

**Results:**

A total of 100 families participated in the study. The median age of the pediatric patients was 5 years (IQR 11.75), and the median age of caregivers was 37 years (IQR 14). Congenital conditions accounted for 65% (n=65) of the underlying diagnoses. Overall, 90% (n=88/98) of the interviewed families expressed interest in telemedicine as an integrative health care solution for their children, whereas only 15% (n=15) reported on previous experiences. A primary telemedical consultation was considered acceptable by 53% (n=50/95) of the participants. Caregivers’ preferences were not significantly associated with distance to the nearest hospital (*P*=.77), employment status (*P*=.89), and family size (*P*=.59).

**Conclusions:**

Caregivers in an urban pediatric surgical setting show substantial interest in telemedical care options. Acceptance appears to be independent of geographic proximity to health care facilities, suggesting that telemedicine may represent a relevant addition to pediatric surgical care even in metropolitan regions. Further studies are needed to evaluate practical implementation, including technical, legal, and compliance-related aspects.

## Introduction

Digital technologies in medicine are being used in various forms of telemedical applications, including the documentation of vital parameters via mobile applications, electronic communication between out- and inpatient sectors, and video consultations [1-4]. In addition, digitalization allows the needs of increasingly empowered and health-conscious patients to be considered [5-8].

However, telemedicine is still underrepresented in pediatric and adolescent medical care, due to the limited experience of practitioners in this field and the associated organizational and logistic challenges [9]. Contact restrictions changed everyday hospital life during the SARS-CoV-2 pandemic, leading to a significant increase in the use of telemedical measures worldwide. A high level of satisfaction was also registered among health care professionals, willing to pursue further innovation [10,11]. Even in mandatory telemedical exchange during the pandemic, patients showed support and acceptance [12]. In the specialty of pediatrics, parents have also expressed a high level of approval and satisfaction with telemedicine measures. The integration of telemedical solutions, particularly within the domain of pediatrics, which is characterized by a youthful demographic, appears to hold considerable potential for enhancing diagnostic and therapeutic processes [13-15]. This is especially important since pediatric and adolescent medicine occupies a unique position. The patients are minors, and thus their interests are inherently represented by their legal guardians; in the context of medical appointments, this can result in double absences from educational institutions and work, which may be accompanied by a loss of earnings and travel expenses [8].

Several studies have demonstrated that telemedical care can yield economic and ecological benefits. Goedeke et al showed that telemedical follow-up in pediatric surgery has been shown to significantly reduce days of absence from school and work, loss of earnings, as well as travel costs and time. A cost analysis by Adroher Mas et al further demonstrated that telemedical follow-up with remote telemonitoring at home for pediatric conditions achieved a 9% reduction in costs compared to inpatient treatment. Moreover, telemedicine can lead to a reduction in the number of visits to the pediatric emergency room and has proven to significantly ameliorate depression and anxiety during hospital admission of both the patients and their parents [16,17]. In addition, telemedicine has been proven to enhance the provision of adequate, specialized medical care in rural areas, where it is known to be notably deficient, even in European countries [2,4,8,15,18-21].

Yet, studies exploring the impact and reception of telemedicine, especially in densely populated regions, are missing. Therefore, the primary objective of this study was to explore the willingness and demand of caregivers for telemedical care in the field of pediatric surgery in a metropolitan area in Germany.

## Methods

### Data Collection and Inclusion Criteria

A 15-item survey was designed by the Department of Pediatric Surgery of the University Medical Center Hamburg-Eppendorf (UKE). The questionnaire (Multimedia Appendix 1) was developed independently in two ways. First, it was developed with regard to clinical practice in our hospital. Second, it was modified on the basis of the extant literature describing the effects of telemedicine on health care and patients [8,13,22-24].

It featured both single-choice and multiple-choice questions, gathering information about the patient’s place of residency, familial, as well as parents’ professional status. The survey inquired about an absence from work, school, and kindergarten to attend the scheduled appointment, as well as participants’ previous experiences with telemedicine and their willingness to engage in telemedical treatment.

Upon informed consent, the questionnaire was distributed during the scheduled presentation in the pediatric surgical clinic, between May and November 2023, and was completed by one of the caregivers.

All patients scheduled for an elective outpatient appointment, whether for initial or follow-up examinations, at the Department of Pediatric Surgery of the UKE were included in the study. Exclusion criteria comprised patients’ or custodians’ language barrier and patients’ age above 18 years. Patients with pediatric trauma conditions were also excluded from the study.

### Ethical Considerations

The study adhered to the principles outlined in the Helsinki Declaration and received approval from the Hamburg Ethics Committee (2023‐101015-BO-ff). The inclusion of patients was carried out after the caregivers had been provided with information about the study and had given voluntary consent. Participants did not receive any financial compensation. Data were deidentified to safeguard participant information. Following the conclusion of the study, the data were anonymized.

### Statistical Analysis

Statistical analysis was carried out using Microsoft Excel for macOS (version 16.59) and Prism 9 for macOS (version 9.5.0). The significance level was set at *P*<.05 for all statistical analyses. Descriptive statistics are presented in total numbers. Continuous variables were checked for deviation from normal distribution (Shapiro–Wilk test) for each comparison. Differences between groups were calculated using the *χ*^2^ test or Fisher exact test. Missing data were excluded from the statistical calculation. A post hoc power analysis was performed to evaluate whether the available sample size (N=100) provided sufficient statistical precision for the primary outcome, defined as the proportion of caregivers expressing interest in telemedicine. Using the observed agreement rate of 90% and a conservative hypothetical reference proportion of 70%, the achieved power to detect such a difference was 99.7% at a two-sided α of .05. This indicates that the study was adequately powered to detect a clinically meaningful deviation from the reference proportion.

The manuscript was written based on the STROBE (Strengthening the Reporting of Observational studies in Epidemiology) cross-sectional checklist [25].

## Results

A total of 100 families participated in the study. The related patients consisted of 69 boys and 31 girls. The median age of the patients was 5 years (IQR 11.75); that of their accompanying custodians was 37 years (IQR: 14). A total of 65% (n=65) of the patients presented with a congenital disorder, ranging from minor pediatric surgical diseases like inguinal hernia to chest wall deformities or major surgical diseases like esophageal or anal atresia, gastroschisis, and Morbus Hirschsprung. In 63% (n=63) of cases, the patients were accompanied by their mother, while in 34% (n=34) of cases, they were accompanied by their father.

Key demographics and sociodemographic characteristics are detailed in Tables1  2.

**Table 1.** Key demographics of patients and caregivers in our clinic’s outpatient pediatric surgery consultation between May and November 2023.

Characteristics	Participants (N=100)
Sex, n	
Female	31
Male	69
Patients age in years (median, IQR)	5 (11.75)
Congenital disorder, n	65
Accompanying person, n	
Mother	64
Father	34
Other	2
Custodians’ age in years (median, IQR)	37 (14)

**Table 2.** Sociodemographic criteria of pediatric surgery patients’ caregivers in our clinic’s outpatient consultation between May and November 2023.

Sociodemographic criteria	Participants (N=100), n
Number of children	
1	36
2-3	50
>3	14
Distance between home and hospital (km)	
<5	15
5-10	21
>10	63
n/a^a^	1
Occupation	
Employed	71
Self-employed	6
Unemployed	14
No information	8
n/a	1
If applicable, absence from^b^	
Work	26
Kindergarten/School	34
Utilization of annual leave	25

a No answer was given.

b Multiple answers allowed; patients and caregivers.

In 90% (n=88/98), the participants expressed an interest in the use of telemedical applications in the treatment of their children, whereas only 15% (n=15) had previous experience with telemedicine. It was found that 53% (n=50/95) of participants would opt for a primary telemedical appointment, under the assumption that the quality of treatment would remain consistent. The existing general treatment process was considered to be sufficiently digitalized (eg, online appointment scheduling or integration of previous examinations and documents) by 57% (n=51/89) of the respondents.

Patients and caregivers were further categorized into two groups based on their median age: children under and over five years old, as well as caregivers under and over 37 years old. No significant differences were observed between these groups regarding the preference for a primary telemedicine appointment.

The results indicated a marginal preference for downloading free applications for telemedical information exchange (n=52/98, 53%). Thirty-eight percent of caregivers opted for a download of fee-based programs. However, 7% (n=7) of participants would not download additional programs for the telemedical treatment of their children.

A substantial proportion of the participants (88%, n=86/98) expressed their willingness to transmit photographic documentation as part of the telemedical information exchange. Concerns regarding adherence to data protection guidelines were documented by approximately one-third of the participants (36%, n=35/98).

Regarding the expressed interest in a primary telemedicine appointment, no statistically significant differences were found regarding selected sociodemographic criteria. A significant increase in the preference for a primary telemedicine appointment was observed only among guardians who consented to downloading paid applications (*P*=.01). The results are displayed in Table 3.

**Table 3.** Results of the sociodemographic and medical criteria of the participating families in relation to a preferred primary telemedical appointment^a^.

Variables	Primarily telemedical counseling, n (%)	No primarily telemedical counseling n (%)	*P* value
Congenital disorder			.96
Yes	32 (33.7)	29 (19)	
No	18 (30.5)	16 (16.9)	
Pre-existing illness			.49
Yes	6 (6.3)	3 (46.3)	
No	44 (3.2)	42 (44.2)	
Questionnaire completed by			.12
Mother	29 (30.5)	31 (32.3)	
Father	21 (22.1)	12 (12.6)	
Other	0 (0)	2 (2.1)	
Number of children			.59
1	15 (15.8)	18 (18.9)	
2-3	27 (28.4)	21 (22.1)	
>3	8 (8.4)	6 (6.3)	
Distance home-hospital (km)			.77
<5	7 (7.4)	8 (8.5)	
5-10	10 (10.6)	10 (10.6)	
>10	33 (35.1)	26 (27.7)	
Occupation of the caregiver			.89
Employed	37 (39.4)	32 (34)	
Self-/Unemployed	13 (13.8)	12 (12.8)	
Day of absence			
Work			.39
Yes	15 (15.8)	10 (10.5)	
No	35 (36.8)	35 (36.8)	
Vacation day			.65
Yes	14 (14.9)	11 (11.7)	
No	35 (37.2)	34 (36.2)	
Kindergarten/School			.81
Yes	17 (18.1)	16 (17)	
No	33 (35.1)	28 (29.8)	
Download of applications			
Free of charge			.33
Yes	24 (25.8)	26 (28)	
No	25 (26.9)	18 (19.4)	
Fee-based			.01^b^
Yes	25 (26.9)	11 (11.8)	
No	24 (25.8)	33 (35.5)	
Concerns about data protection			.24
Yes	14 (14.9)	18 (19.2)	
No	35 (37.2)	27 (28.7)	

a Missing data were excluded from the statistical calculation.

b Statistical significance (*P *value<.05).

## Discussion

### Principal Findings

This survey showed a high level of interest among the patients’ caregivers in the implementation of telemedicine in pediatric surgery in Hamburg, a metropolis with a population of nearly two million people and a high density of medical professionals [26].

This finding is particularly significant in the context of current literature on telemedicine in pediatrics, which often focuses on providing medical expertise in rural areas [2,4,15,18-21]. Moreover, the present findings corroborate the favorable patient feedback concerning telemedicine documented in the existing literature [10,11]. However, a mere 15% of the participants stated previous experience with telemedicine, underscoring the necessity for additional research in order to establish and advance digitalization in these specialist areas.

Despite the overmentioned high level of interest, the results indicated that only slightly more than 50% of caregivers would select a primary telemedical consultation, even under the assumption that the standard of treatment would remain equivalent. It is thus reasonable to conclude that personal contact with the treating physician still plays a relevant role in pediatric medicine nowadays [14].

The influence of sociodemographic factors on families’ preference for a primary telemedical appointment (given equivalent quality to an in-person one) was analyzed. It is interesting to note that no significant differences were identified among subgroups defined by the distance between the subjects’ place of residence and the hospital. This suggests that the proximity to the nearest hospital does not influence the preference for primary telemedical treatment within the studied population. In previous studies, it has been displayed that telemedical treatment has the capacity to mitigate patients’ loss of work and wages, as well as less travel time [8,27]. A longer journey is generally more time-consuming, which could lead to the assumption that a telemedical appointment should be favored by those participants who live further away from the hospital [28].

Also, the number of children in a family did not impact caregivers’ desire to receive a primary telemedical consultation. 64% of the participants had more than one child; as it has been shown that mothers of two children have higher parenting stress than those with one child, we initially assumed that telehealth consultation would be preferred by those with more children [29]. Luchtenberg et al showed that the child-doctor relationship is characterized by intimacy and trust, which may not be fully achieved through virtual interaction [30]. When implementing telemedicine in pediatric medicine, it is therefore essential to establish a trusting and secure interaction between patient, their caregivers, and health care providers [31].

Furthermore, our results did not show a significant preference for telehealth consultation based on the caregiver’s employment status. Again, one could argue that, for example, self-employed caregivers are more dependent on their free time, therefore more likely to prefer a quicker, non-personal encounter. On the contrary, employed custodians showed a similar willingness compared to self-employed and non-employed ones.

Nearly 70% of the patients were accompanied to their appointments by female caregivers. This confirms a recent study which found that mothers are significantly more likely to be in charge in the cases of children’s medical appointments, both scheduled and not [32]. However, it should be noted that the questionnaire was only completed by one caregiver, despite the possible presence of both parents at the appointment. Consequently, it cannot be definitively established whether most patients were accompanied only by their mothers.

Regarding the question of whether patients’ caregivers would be willing to pay for a medical application, the findings of our study indicated a slight majority of 53% opting for free applications instead of paid ones. Existing instant messaging applications with a video-call function represent a viable option in this regard, on the condition that they follow data protection laws. Since approximately 90% of all participants would be willing to transmit photographs, this is of even greater significance.

### Limitations

It is imperative to acknowledge certain limitations in our study. The analysis did not consider the rejection rate, which could have provided a more comprehensive overview of general participation in the study, which is therefore susceptible to selection bias [33]. The documentation of possible reasons why legal guardians refused to participate could also have provided greater insight.

Due to the limited number of 100 participants, our results may not be representative, but rather provide a preliminary overview of the subject. In addition, as a university hospital and tertiary referral center, our patients’ spectrum is not fully comparable with that of other primary and secondary referral centers in Hamburg, located in other areas of the city. Moreover, the collective comprises a highly heterogeneous selection of patients who presented with a variety of diseases both pre- and postoperatively.

Caregivers of patients with other underlying conditions may have responded differently to our questionnaire. Further, the present study did not investigate whether different socio-economic or cultural aspects might influence the general willingness to use telemedicine in pediatric surgery [34]. A further potential limitation is the distribution period, which may be subject to a temporal or seasonal bias.

Methodically, our questionnaire contained only 15 items; it served as a cursory opinion survey, on whose basis more detailed follow-up studies can be tailored.

As previously stated, a mere fraction of the study’s participants had prior experience with telemedical applications. This limited experience may hinder one’s ability to envision how a telemedicine consultation would occur and whether it could match the quality of an in-person encounter. Anyway, the study population accurately reflects the current patient pool at our clinic, thereby ensuring the validity of the findings. The external validity of the study results applies only to a limited extent here, as country-specific regulations and infrastructure can have a decisive influence on the use of telemedicine.

### Conclusion

In conclusion, the potential use of telemedicine in the treatment of pediatric surgical patients in urban areas and cities is perceived and viewed positively and is regarded as a welcome addition. We were able to show that a significant majority of caregivers had considerable interest in utilizing such measures, even in a big city with a high density of health care providers. This could be established through various means, including video consultations, preoperative consultations, post-operative follow-up checks with photographic documentation, and the further integration of artificial intelligence. The implementation of these measures has, for example, the potential to result in a reduction of visits to pediatric emergency departments or to support the transition of pediatric surgical patients to adult medicine [16,35,36]. In this context, it is essential to ensure that reimbursement by health insurance companies is guaranteed in order to offer telemedicine measures on a generalized and sustainable basis [37].

However, the study also evidenced that telemedicine does not play a significant role in the current day-to-day pediatric surgical treatment in Hamburg, the second largest city in Germany.

The necessity for the routine establishment of telemedical infrastructures is thus apparent. Further studies may elucidate how to correctly implement telemedicine in pediatric surgery, for example, by documenting parents’ and practitioners’ compliance as well as listing technical and legal obstacles.

## Supplementary material

10.2196/81091 Multimedia Appendix 1 Questionnaire.

10.2196/81091 Checklist 1 Completed STROBE checklist (where applicable).

## Abbreviations

STROBE: Strengthening the Reporting of Observational Studies in Epidemiology
UKE: University Medical Center Hamburg-Eppendorf
