# Supplementary material for: Parental Acceptance of Telemedicine in Pediatric Surgery and Its Implications for Future Care Models: Survey Study
Source: JMIR Form Res. 2026 Feb 23;10:e81091. doi: 10.2196/81091 (PMC12928718; doi:10.2196/81091)
Supplement: Multimedia Appendix 1 [file formative-v10-e81091-s001.docx]

|  |
| --- |

**Parent Questionnaire "Telemedicine in Pediatric Surgery"**

To be filled out by the healthcare provider:

Name, First Name:

Date of Birth:

**Diagnose:**

- Initial Consultation
- Follow-Up Consultation

To be filled out by the parents/caregivers:

1. How old is your child:
2. How old are you:
3. What is your relationship to the child:

- Mother
- Father
- Other: ___________________________

1. How many children do you have:

- 1
- 2-3
- >3

1. How far is your place of residence from the University Medical Center Hamburg-Eppendorf:

- <5 km
- 5-10 km
- >10 km

1. What is your employment status:

- Employed
- Self-employed
- Currently not employed
- No information

1. Do you use mobile devices (smartphone/laptop/tablet) at home?

- Yes
- No

1. Were there any of the following circumstances for the current appointment (multiple answers possible)?

- Work absence
- Vacation day
- Absence from school/kindergarten?

1. Do you have any experience with telemedicine in medical treatment (e.g., apps, video consultation):

- Yes
- No

1. Are you generally interested in using digital solutions for your child's treatment (e.g. with an application, via email or video call)?:

- Yes
- No

1. Would you be willing to download specific apps or programs for this purpose?

- Yes, including paid apps
- Yes, only apps free of charge
- No

1. Would you be willing to send photos via the telemedicine application (e.g. via a messaging service):

- Yes
- No

1. Would you have concerns regarding data protection compliance in the event of telemedicine treatment?

- Yes
- No

1. Would you prefer a telemedicine appointment over an in-person visit to the doctor (assuming the quality of treatment is the same; e.g. as part of a planned follow-up):

- Yes
- No

1. Is your child’s general treatment process in hospital sufficiently digitized (e.g. appointment scheduling, integration of preliminary examinations or documents):

- Yes
- No
